# Effects of different oral barrier membranes on the efficacy and safety of guided bone regeneration in patients with dental implants: a systematic review and meta-analysis

**DOI:** 10.2340/aos.v84.43758

**Published:** 2025-06-10

**Authors:** Haozhou Tang, Yonghui Zhang

**Affiliations:** a Department of Stomatology, Xingyi People’s Hospital, Xingyi, China; b The Affiliated Stomatology Hospital of Kunming Medical University, Kunming, China

**Keywords:** absorbable oral barrier membrane, non-absorbable oral repair membrane, dental implant, guided bone regeneration

## Abstract

**Objective:**

This study aims to systematically evaluate the effects of different oral barrier membranes on bone regeneration, focusing on their clinical efficacy and safety during dental implant procedures.

**Methods:**

A comprehensive search was conducted in PubMed, EMBASE, ScienceDirect, Cochrane Library, CNKI, VIP, and CBM databases for case-control and cohort studies published between January 2002 and March 2025. Two independent researchers screened and extracted data, and statistical analysis was performed using RevMan 5.3. The study was registered in PROSPERO (CRD492390).

**Results:**

A total of 11 clinical controlled and cohort studies with 1,003 patients were included. The absorbable membrane group demonstrated a significantly higher success rate (*p* < 0.05), greater bone graft thickness (*p* < 0.05), and fewer adverse reactions (*p* < 0.05). Meta-analysis showed no significant difference in osseointegration, total mineralised tissue, and non-mineralised tissue (*p* > 0.05).

**Conclusion:**

Absorbable oral barrier membranes exhibit superior safety and efficacy profiles, making them a preferred choice for guided bone regeneration. However, further studies with higher methodological quality and longer follow-up durations are required.

## Introduction

Dental implants have become a widely recognised treatment for dental defects and missing teeth, offering a safer and more effective therapeutic option [1, 2]. However, the success of these treatments is closely tied to addressing challenges such as insufficient bone volume, which can impact the outcomes of implant surgeries. Successful dental implant placement requires adequate healthy bone surrounding the implant [1]. Dental implants consist of biocompatible artificial roots implanted in the area of missing teeth [3]. Over time, these roots integrate with the alveolar bone, achieving osseointegration [4]. A crown is then added to restore functionality, making implant restoration a preferred option because of its minimal impact on adjacent teeth, ease of use, strong support, and effective masticatory function [5].

Despite advancements in implant technology, implant loosening or shedding is sometimes observed in clinical practice [6]. Factors contributing to these issues include implant material, design, surgical techniques, and postoperative maintenance. However, a significant cause is insufficient alveolar bone in the edentulous area, highlighting the importance of ensuring adequate bone for long-term implant success [7–9]. The design of dental implants significantly influences their stability and overall success, affecting their ability to withstand occlusal forces and integrate with the surrounding bone [10].

Guided Bone Regeneration (GBR) is a vital technique that addresses the problem of insufficient bone volume by using a barrier membrane to protect the bone defect area. This creates a conducive environment for precursor osteoblasts to grow and remodel, ultimately promoting bone regeneration [11]. This technique originated from studies on cellulose acetate in bone defect healing in the 1970s [12]. GBR has been shown to increase alveolar bone volume significantly, enhancing osseointegration and implant stability [14–17]. The surgical process for GBR starts with assessing the patient’s bone condition and exposing the bone defect area via an incision. Bone grafts and a barrier membrane – either resorbable or non-resorbable – are applied to support bone regeneration [18]. Non-resorbable membranes like titanium require surgical removal, while resorbable membranes such as collagen degrade naturally, eliminating the need for additional surgery [19, 20].

Barrier membranes are fabricated using advanced technologies to ensure precision and adaptability. Non-resorbable titanium membranes are often laser-cut, while resorbable membranes, primarily derived from animal collagen, are processed to retain structural integrity for effective bone regeneration before biodegrading [21, 22].

The material in GBR is mainly composed of bone material and barrier membrane. At present, there are many types of GBR barrier membranes in the market. According to their biodegradability, they can be divided into two types, namely absorbable membranes and non-absorbable membranes. Due to their different physical and biological properties, these two types of barrier membranes have both advantages and disadvantages [23]. In particular, the non-resorbable membrane, represented by the titanium membrane, can continue to act as a mechanical barrier to the surrounding tissue in the patient’s body, providing sufficient time and space for the regeneration of bone tissue. At the same time, it is easy to manipulate and the time it remains in the body depends on the size and type of bone defect. However, its biggest disadvantage is that it does not avoid secondary surgical incisions and can lead to mucosal wounds because of poor histocompatibility. The risk of dehiscence, membrane exposure, and wound infection can affect bone formation [24]. The absorbable membrane represented by collagen membrane is a kind of xenogeneic acellular matrix material. Because it can absorb itself, it does not need to be taken out twice [25]. The collagen membrane itself is resistant to infection after exposure and the probability of wound infection is minimal [26, 27]. Resorbable collagen membranes have reduced collagen due to differences in cross-linking techniques and processing. These membranes are the preferred choice for GBR because of their improved biocompatibility and controlled degradation rate. Composed mainly of collagen, they remove cellular components through bioengineering technology and retain the extracellular matrix and dermal scaffold for cell migration. They promote tissue growth, wound healing, and prevent immune rejection reactions [28, 29].

In recent years, dental implants have been used as a reliable method for restoring missing teeth. The success of this method depends on having sufficient healthy bone at the implant site; however, bone volume deficiency can lead to issues such as implant loosening or failure [30]. The GBR technique, using both absorbable and non-absorbable membranes, creates an appropriate space for bone restoration. Absorbable membranes, such as collagen membranes, have gained popularity due to their lack of need for secondary surgery and reduced risk of infection. Platelet-Rich Fibrin (PRF) membranes have gained attention in GBR because of their autologous nature and bioactivity. Recent studies have demonstrated that different PRF formulations exhibit distinct mechanical and structural properties.

Various studies have examined the effectiveness of absorbable and non-absorbable membranes in bone regeneration, but the results have been inconsistent and evaluations have varied. Therefore, high-quality research evidence is essential to determine the clinical value of these membranes. This systematic review and meta-analysis aims to evaluate the efficacy and safety of absorbable versus non-absorbable barrier membranes for bone regeneration in dental implants, providing objective information for clinical application and future research.

## Methods and materials

The PRISMA 2020 approach for Systematic Review (PRISMA-SR) was used to conduct this study [31].

### Documents sources and retrieval methods

We used several search engines and databases such as PubMed, EMBASE, ScienceDirect, Cochrane Library, CNKI, VIP full-text Database, CBM, Chinese and foreign periodicals, conference papers, degree papers to obtain the pertinent data. This systematic review followed the recommendations of PRISMA (Preferred Reporting Items for Systematic Review and Meta-Analyses). We registered this study prospectively in PROSPERO with registration number CRD492390. The relevant data of case-control trials or cohort trials on the clinical efficacy of absorbable and non-absorbable oral barrier membranes in guiding bone regeneration in dental implants were collected.

### Literature inclusion and exclusion criteria

#### Inclusion criteria

Study types: research on clinical efficacy of absorbable and non-absorbable oral barrier membranes in guiding bone regeneration during dental implant placement [2]. Subjects: (1) patients who chose implant denture repair because of dentition defect without implant contraindication, regardless of age, sex, or race; (2) Patients with bone defect in edentulous area needed GBR to repair bone defect; (3) Interventions: appropriate absorbable oral barrier membrane was placed in the bone graft area to guide bone regeneration, while in the control group, appropriate non-absorbable oral barrier membrane was placed in the bone graft area to guide bone regeneration; (4) More than one outcome indexes: repair, incidence of adverse reactions and complications, bone thickness, bone graft thickness, histological level analysis, and so on.

#### Exclusion criteria

(1) Studies other than case-control and cohort studies such as cross-sectional, descriptive, qualitative studies, among others; (2) There were errors in data report, which prevented data from being used; (3) Research content that was duplicated was selected based on the most recent study; (4) Neither the study’s curative effect nor its effectiveness was remarkable; (5) Review; and (6) Clinical cases.

### Search strategy

The literature was searched by keywords of absorbable oral barrier membrane; non- absorbable oral barrier membrane; dental implant; GBR; systematic review; meta-analysis from January 2002 to March 2025.

### Selection process

**Figure 1:**
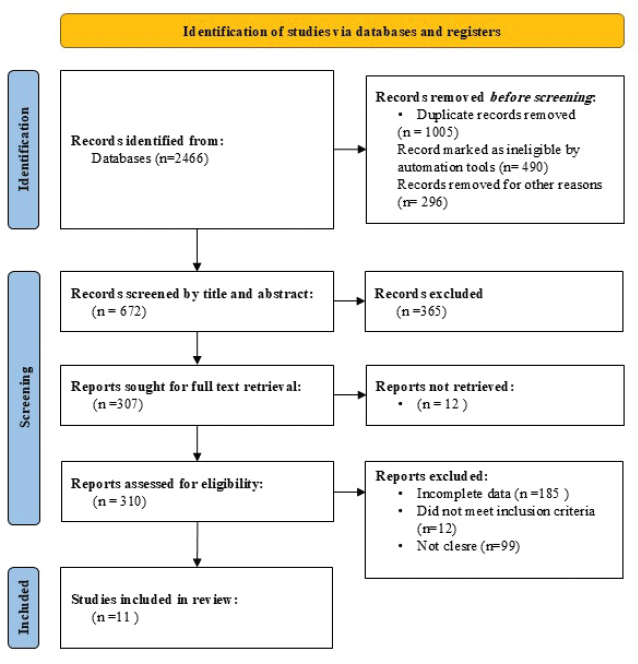
Study flow chart.

At the outset, one of the researchers transferred all search results from the databases into EndNoe Desktop software, removing any duplicates. Following this, two researchers independently evaluated the titles and abstracts of the articles according to established eligibility criteria. If there were any disagreements regarding study selection, a comprehensive review of the full text was performed, with a third researcher involved if needed. Efforts were made to obtain inaccessible articles and unpublished data by contacting the corresponding authors of the eligible studies. The screening process was visually represented using the PRISMA 2020 flow diagram [31] (Figure 1).

### Quality evaluation and data extraction

1. Bias risk assessment: The quality of the studies was evaluated using the Cochrane Risk of Bias tool, which assesses bias across seven domains: random sequence generation, selective reporting, blinding of participants, blinding of personnel, blinding of outcome assessment, allocation concealment, and incomplete outcome data. The tool evaluates the risk of bias for each domain as low, unclear, or high risk. It is important to note that blinding of participants and blinding of personnel are considered as separate domains in this tool. In addition, the ‘other types of bias’ domain includes a variety of potential biases not covered by the other six domains, such as conflicts of interest, early termination of studies, or funding bias [32].
2. Literature screening and data extraction: Two researchers screened literature, gathered data, assessed results quality, and crossed-checked findings. In case of disagreement, discussions were held to resolve or a third researcher assisted in judgement. The author was contacted if the data were incomplete. Data extraction contained: (a) author, publication time, number of cases; (b) scheme, course of treatment; (c) recurrence, adverse reactions and incidence of complications; bone thickness, bone graft thickness, histological level analysis, and other indicators (Table 1).
3. Statistical processing: Meta analysis was performed using RevMan5.3 software. Counting data were analysed using relative risk (RR) and continuous data were analyzed using mean difference (MD). Each effect given point estimate and 95% confidence interval (CI). *I*^2^ and the χ^2^ test assessed the heterogeneity in the meta-analysis. Heterogeneity in the data can be analysed through subgroup, sensitivity, or descriptive analysis. Fixed effect model can be adopted if there is no heterogeneity, and a random effect model can be adopted if the analysis exhibits heterogeneity. The significant value was set at p < 0.05. Eggers’s test was used to examine the funnel chart’s asymmetry. An inverted funnel chart analyzes literary publication bias. Trimand Fill method correct funnel chart whenever *p* value is less than 0.1, thereby adjusting release deviation effect.

**Table 1:** Basic characteristics of included studies.

Study (Year)	Country	Inclusion/ exclusion criteria	Sample size		Intervention measures		Research time limit	Grouping method	Adverse effect	Outcome index
			C group	R group	C group	R group				
He Liming (2017) [33]	China	Patients were required to undergo single-tooth dental implant procedures with bone regeneration guidance. The age range of participants was 18–66 years. Exclusion criteria: Patients with active infections within 1 week before surgery. Pregnant or lactating women. Patients with specific allergic constitutions, especially those allergic to collagen-based materials.	110	110	Non-absorbable film	Absorbable film	2014.5–2015.6	Random drawing of lots	Observation group (Hai’ao Membrane): Local swelling: 3 cases Wound dehiscence: 3 cases Total adverse events: 6 cases (5.45%) Control group (Titanium membrane): Local swelling: 6 cases Wound dehiscence: 8 cases Total adverse events: 14 cases (12.73%)	➀➁➂
Hou Yuyi (2017) [34]	Germany	The participants’ ages ranged from 27 to 70 years. Exclusion Criteria: Patients with severe systemic diseases (e.g., liver failure, kidney failure, or cardiopulmonary disorders). Patients who were unable to follow up and complete the study. Patients with severe inflammatory issues or acute oral infections, which could interfere with study outcomes	34	34	Non-absorbable film	Absorbable film	2015.4-2016.8	not clear	Titanium Membrane Group: Wound dehiscence: 6 cases (26.47%) Facial swelling: 3 cases Total adverse events: 9 cases (26.47%) Oral Prosthetic Membrane Group: Wound dehiscence: 1 case (5.88%) Facial swelling: 0 cases Total adverse events: 2 cases (5.88%)	➀➁➂
Wang Lu (2018) [35]	China	Inclusion Criteria: Patients aged 20 and above with single-tooth loss, actively cooperating in the study. Exclusion Criteria: Severe liver and kidney diseases. Severe underlying conditions or systemic infections. History of mental illness or communication disorders.	35	35	Non-absorbable film	Absorbable film	2016.2-2017.5	not clear	Observation group: 2.86% (1 case of wound dehiscence). Control group: 11.43% (2 cases of acute swelling, 2 cases of wound dehiscence).	➀➁➂
Guo Yanjie (2018) [36]	China	Inclusion Criteria: Patients aged 18 to 66 requiring bone augmentation for dental implants. No infection in the past week. Exclusion of pregnant or breastfeeding women and individuals with collagen allergies. Exclusion Criteria: Infection within the past week. Pregnancy or breastfeeding. Specific allergies, especially to collagen.	83	74	Non-absorbable film	Absorbable film	2016.3-2017.6	Random number table method	Observation group: 6.36% (including local swelling and cracking). Control group: 11.8%.	➀➁➂
Jin Gaojie (2017) [37]		Inclusion Crite-ria: Periodontally healthy patients (probing depth <4 mm). Good oral hy-giene (plaque index <25%). Adequate in-flammation control (bleed-ing on probing <25%). Single-tooth gaps with buc-cal alveolar bone deficien-cy. Implant place-ment planned at least 6 weeks after tooth ex-traction. Presence of a dehiscence or fenestration-type defect after implant place-ment. Exclusion Crite-ria: Patients who did not meet the inclusion crite-ria were exclud-ed before sur-gery. If no bone augmentation was needed af-ter implant placement, the patient was ex-cluded from the study.	42	42	Non-absorbable film	Absorbable film	2014.8-2015.11	not clear	Soft tissue dehiscence: 30% in the resorbable membrane (RES) group and 14% in the non-resorbable membrane (N-RES) group. Loss of horizontal bone thickness: 2.23 mm in the RES group, whereas only 0.14 mm in the N-RES group (statistically significant difference). Vertical defect resolution: 85% in the RES group and 90.7% in the N-RES group. No impact of membrane exposure on final outcomes: In cases where membrane exposure occurred, infection was controlled, and early membrane removal was not required.	➀➁➂
Friedmann (2002) [38]		Inclusion: Pa-tients had to be 18 years or old-er. Candidates had to require dental implant place-ment in the mandible or maxilla. Primary stabil-ity of the im-plant must have been achieved in a single-stage procedure. A minimum vertical defect height of 4 mm had to be pre-sent at the time of implant placement. Women of childbearing potential had to have a negative pregnancy test and agree to contraception for 6 months post-surgery. Patients had to be able to com-ply with study procedures and provide written informed con-sent. 2. Exclusion Criteria: Heavy smokers (>20 ciga-rettes/day). Insulin-dependent dia-betics. Patients with general contra-indications for dental or surgi-cal treatments. Patients with a history of ma-lignancy, radio-therapy, or chemotherapy within the past 5 years. Pregnant or nursing women. Patients using medications that affect bone turnover or mu-cosal healing. Patients with bone metabo-lism disorders or connective tissue diseases. Patients with active infections at the implant site. Patients with limited blood supply to the implant area. Patients with substance abuse issues. Patients in-volved in an-other clinical trial. Patients who had previous treatments with similar materi-als at the same implant site. Insufficient ke-ratinized tissue at the implant site (<4 mm width).	14	14	Non-absorbable film	Absorbable film	not clear	not clear	Test Group (PLGA Membrane): Soft tissue dehiscence/fenestration: 5 cases (26.3%) Membrane exposure: Occurred in some patients, with spontaneous healing in a few cases. Bone augmentation failure: Observed in some patients with soft tissue complications. Control Group (ePTFE Membrane): Soft tissue dehiscence/fenestration: 2 cases (9.5%) Membrane had to be explanted in some cases due to complications.	➃
Naenni (2017) [39]		Inclusion Criteria: Patients aged > 18. No infection in the past week. Exclusion of pregnant or breastfeeding women and individuals with collagen allergies. Exclusion Criteria: Infection within the past week. Pregnancy or breastfeeding. Specific allergies	16	11	Non-absorbable film	Absorbable film	not clear	Computer random	Not reported	➃
Schneider (2014) [40]		Inclusion Criteria: Patients aged > 18. No infection in the past week. Exclusion Criteria: Specific allergies	19	21	Non-absorbable film	Absorbable film	not clear	Computer random	Not reported	➃
Yu Miao (2015) [41]		Inclusion Criteria: Patients aged > 18. No severe physical or mental illnesses. Exclusion Criteria: History of severe systemic diseases. Mental illnesses	53	54	Non-absorbable film	Absorbable film	2010.3-2014.3	Random lottery	Not reported	➀➁➂
He Tianrong (2015) [42]	China	Inclusion Criteria: Patients aged 20 to 68 undergoing dental implant surgery. No severe heart, lung, kidney diseases, infections, or mental illnesses. Exclusion Criteria: History of severe systemic diseases. Mental illnesses or communication disorders.	49	49	Non-absorbable film	Absorbable film	2013.5-2014.3	Random number table method	Observation group: 2.0% (1 case of wound infection). Control group: 8.2% (including facial swelling and wound infection).	➀➁➂
Liu Maofu (2017) [43]		Inclusion Criteria: Patients aged > 18. No severe systemic disease. Exclusion Criteria: History of severe systemic diseases. Mental illnesses	52	52	Non-absorbable film	Absorbable film	2014.2-2016.2	Random number table method	Not reported	➀➁➂

Note:➀Repair condition (success rate of implant operation);➁Incidence of complications or adverse reactions;➂Bone thickness, bone graft thickness;➃Histological level analysis.

### Management of missing data and data preparation

In cases where summary statistics or critical data points were missing, the study authors were contacted to retrieve the necessary information. If the missing data could not be obtained, statistical methods such as imputation or exclusion of the incomplete study were employed, depending on the extent and relevance of the missing data to the analysis. For data preparation, all outcome measures were standardised to ensure consistency across studies. Continuous data, such as mean and standard deviation, were converted to a common unit where necessary. In addition, effect sizes such as odds ratios and risk ratios were transformed into a unified format to facilitate meta-analysis using RevMan 5.3 software.

## Results

### Literature retrieval and basic conditions of literature inclusion

In total, 2,423 articles were retrieved, 1,138 articles after excluding duplicate articles were retrieved. Irrelevant studies, reviews, and case reports, 652 articles were identified, as well as non-controlled studies and non-cohort studies were excluded. Approximately 302 articles were contained, and each article full text was carefully reviewed. A total of 291 articles were excluded because their data were incomplete and they did not include primary outcome indicators. Finally, 11 clinical controlled studies and cohort studies were contained, with 1,003 samples for meta-analysis (Table 1). A diagram showing how the literature screening process works and a table showing basic characteristics of literature (Figure 1).

### Quality assessment of literature-based methodologies

**Figure 2:**
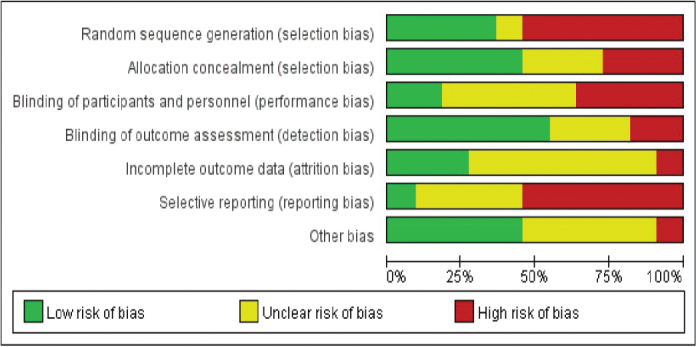
Risk bias chart.

**Figure 3:**
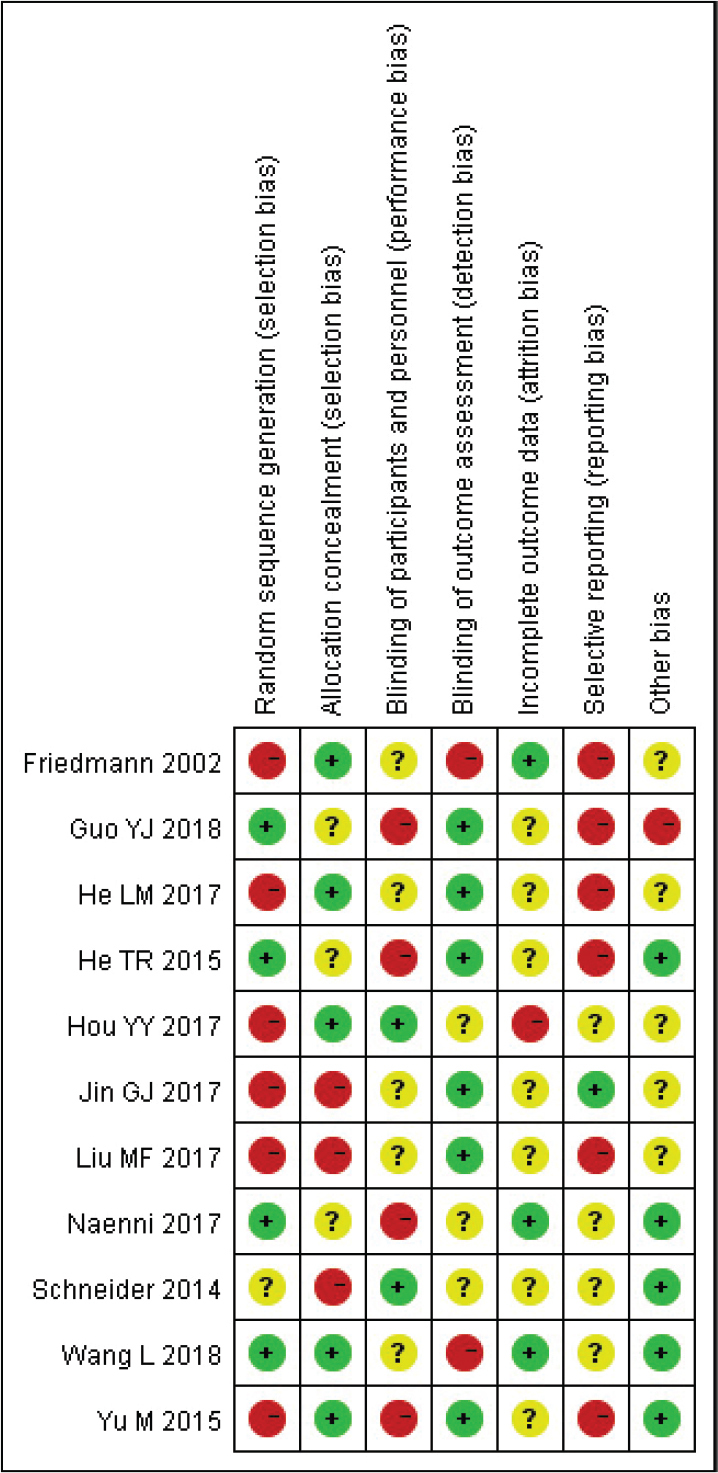
Summary chart of risk bias.

Eight case-control or cohort studies that report baseline patient status were included. In all eight studies, detailed intervention measures and follow-up times were described; however, four papers did not refer to the random method. There was no detailed description of the reasons behind blind methods and withdrawals or loss of follow-up or follow-ups in any of the literatures. According to the Jadad scale, ≥ 3 score indicated high-quality literature, ≤ 2 indicated low-quality literature. The risk bias analyses are displayed in Figures 2 and 3.

### Meta analysis result

#### Success rate of repair

**Figure 4:**
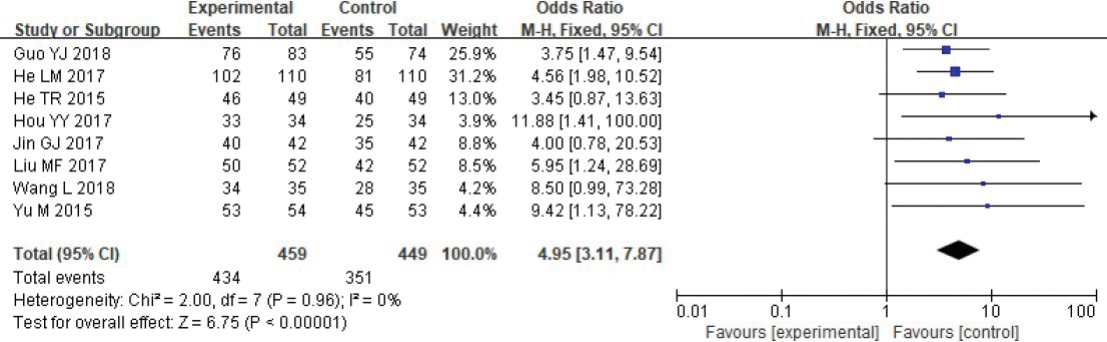
Forest analysis map of comparison of restoration success rates between the two groups.

Meta-analysis was performed on repair success rates. Heterogeneity testing revealed the data contained in this study did not exhibit remarkable heterogeneity: Chi^2^ = 2.00, df = 7, *p* = 0.96, *I*^2^ = 0%. The fixed effect model analyses found (Figure 4) that the absorbable group repair success rate was higher (*p* < 0.05).

#### Thickness of bone graft

**Figure 5:**
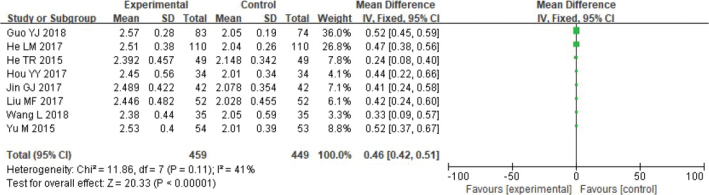
Forest analysis map of comparison of bone graft thickness between the two groups.

Several meta-analyses have been done on bone graft thickness. Heterogeneity testing revealed the data did not exhibit remarkable heterogeneity: Chi^2^ = 11.86 df = 7, *p* = 0.11, *I*^2^ = 41%, and the fixed effect model was used to analyse the data (Figure 5).

#### Osteogenic thickness

**Figure 6:**
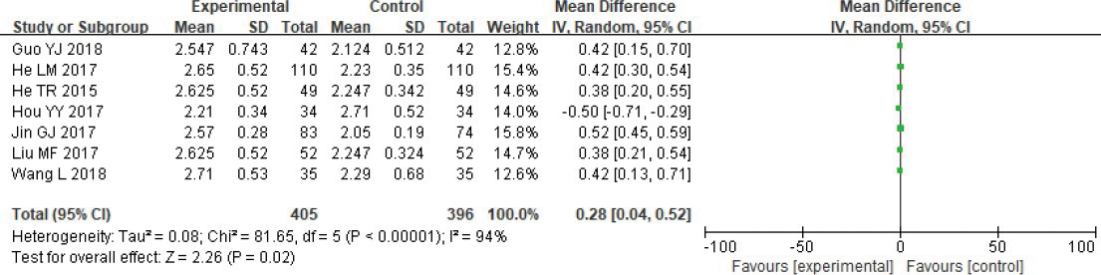
Forest analysis diagram of bone thickness comparison between the two groups.

Meta-analysis was conducted on bone thickness. Heterogeneity testing revealed the data have no remarkable heterogeneity: Chi^2^ = 81.65, df = 5, *p* < 0.00001, *I*^2^ = 94%. Based on the Random effect model, absorbable group’s bone thickness was higher (*p* < 0.05) (Figure 6).

#### Histological level

**Figure 7:**
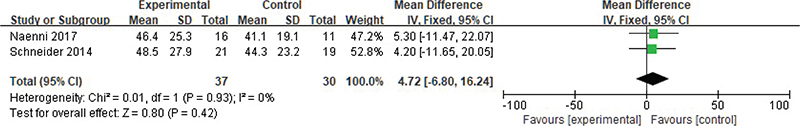
Forest analysis map of comparison of bone union between the two groups.

**Figure 8:**
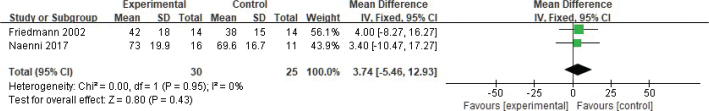
Forest analysis map of the comparison of the total amount of mineralised tissue between the two groups.

**Figure 9:**
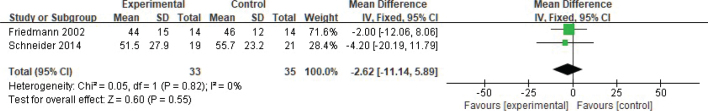
Forest analysis map for comparison of the total amount of two groups of non- mineralised tissues.

Meta-analysis was conducted on histological levels, including osseointegration, total mineralised tissue and total non-mineralised tissue. Heterogeneity test results revealed the following results – osseointegration: Chi^2^ = 0.01, df = 1, *p* = 0.93, *I*^2^ = 0%; total mineralised tissue: Chi^2^ = 0.00, df = 1, *p* = 0.95, *I*^2^ = 0%; and total non-mineralised tissue: Chi^2^ = 0.05, df = 1, *p* = 0.82, *I*^2^ = 0%, indicating the inclusion exhibited no obvious heterogeneity between the study data. Using a fixed effect model analysis, it was found that there was no statistical difference between the osseointegration and the total amount of mineralised tissue and non-mineralised tissue (*p* > 0.05) (Figures 7-9).

#### Safety analysis

**Figure 10:**
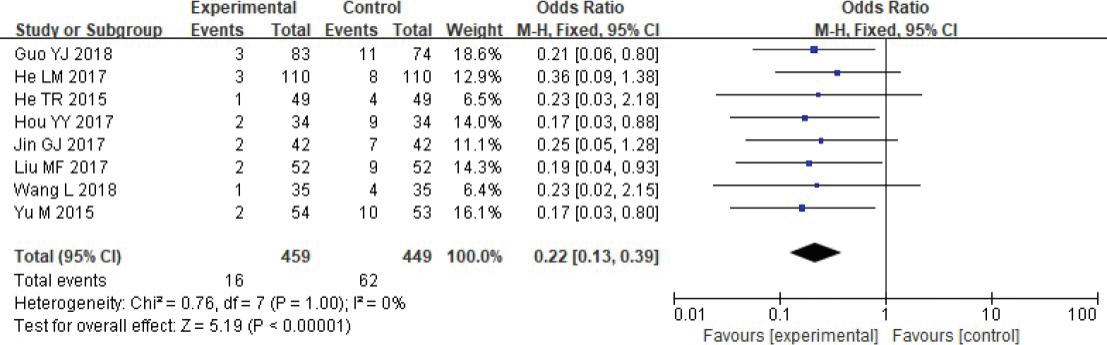
Forest analysis map of the comparison of adverse reaction rates between the two groups.

Adverse reactions were analysed through meta-analysis. Heterogeneity testing revealed the following results: Chi^2^ = 0.76, df = 7, *p* = 1.00, *I*^2^ = 0%, which indicated no remarkable heterogeneity. Fixed-effect model analysis indicated absorbable group had remarkably fewer adverse reactions (Figure 10).

### Publication bias analysis

**Figure 11:**
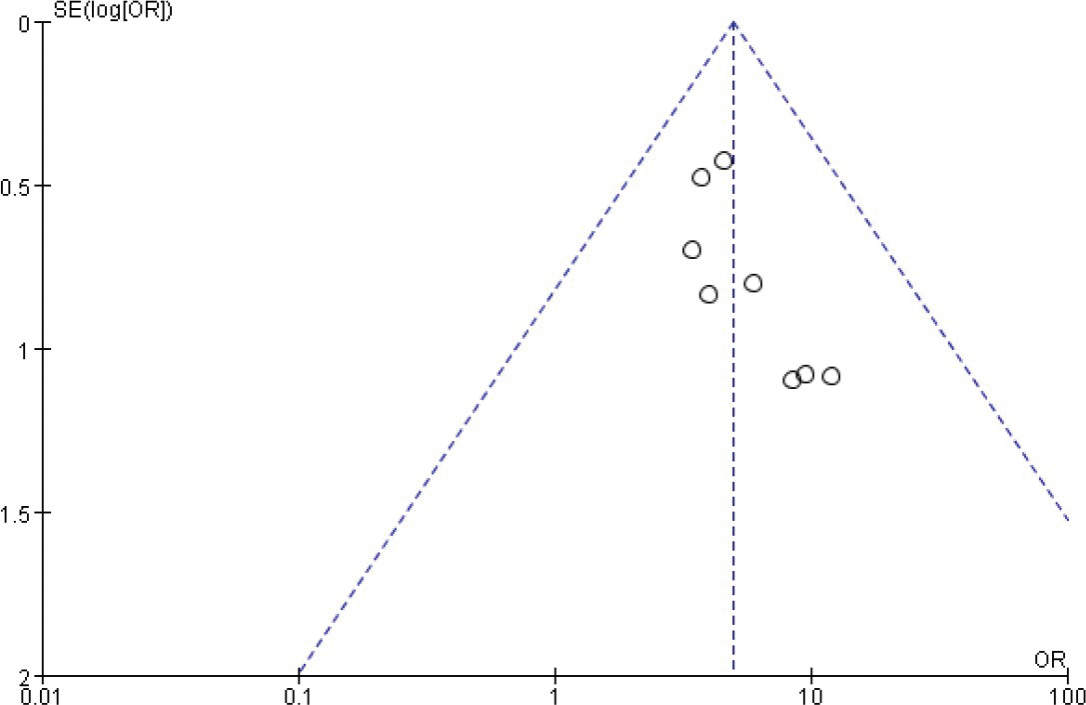
Funnel diagram based on the success rate of restoration.

**Figure 12:**
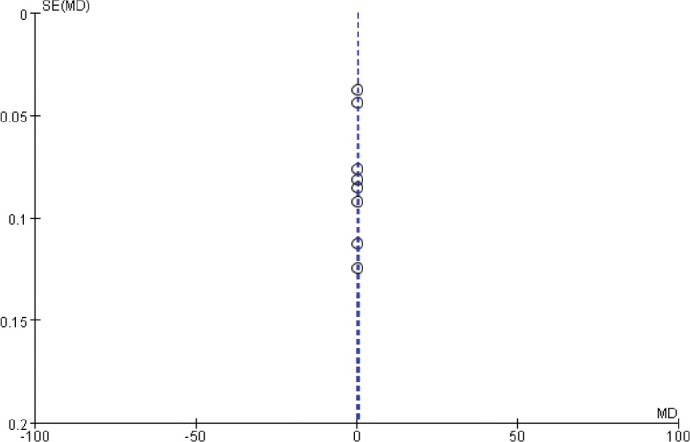
Funnel diagram based on the thickness of bone graft.

**Figure 13:**
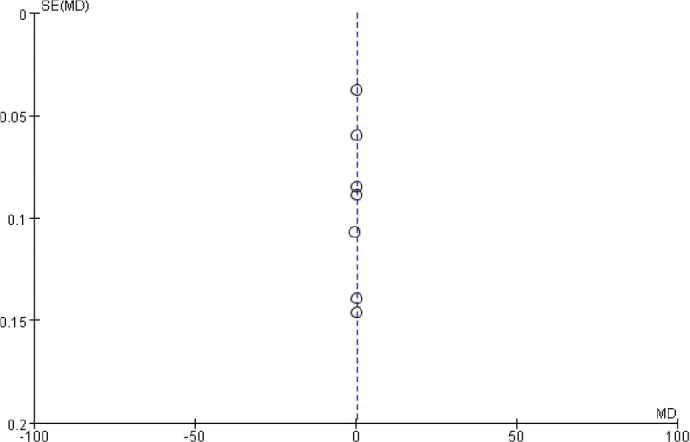
Funnel diagram based on osteogenic thickness.

**Figure 14:**
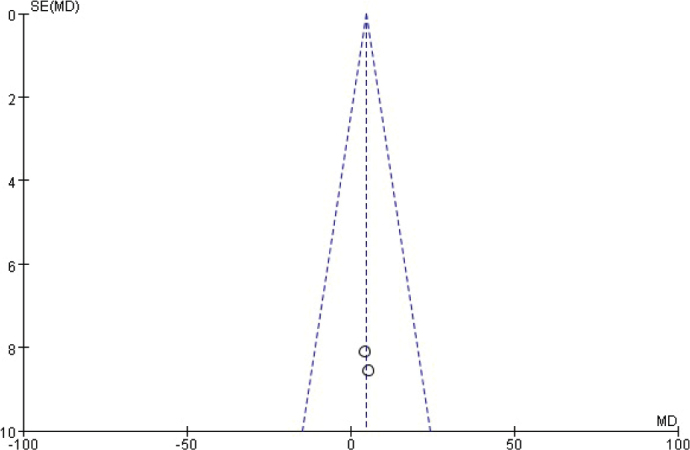
Funnel diagram based on osseointegration.

**Figure 15:**
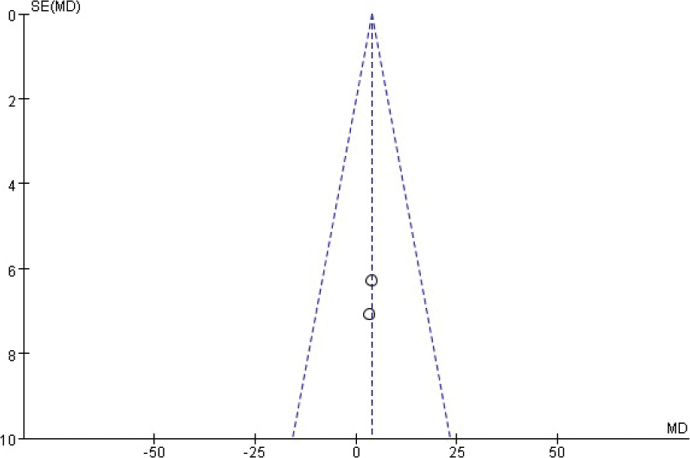
Funnel chart based on the total amount of mineralised tissue.

**Figure 16:**
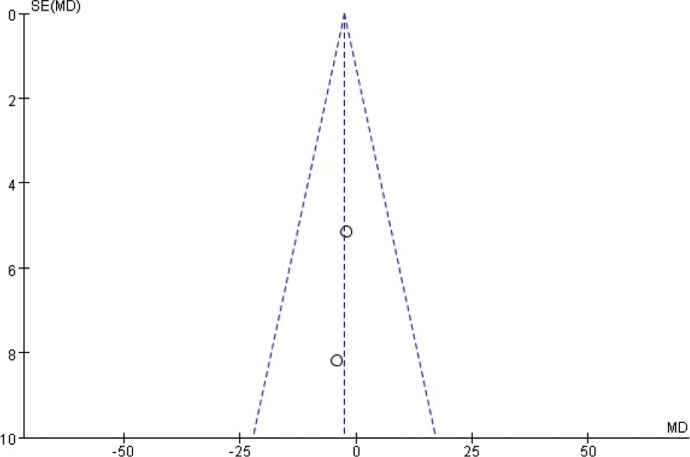
Funnel chart based on the total amount of non-mineralized tissue.

**Figure 17:**
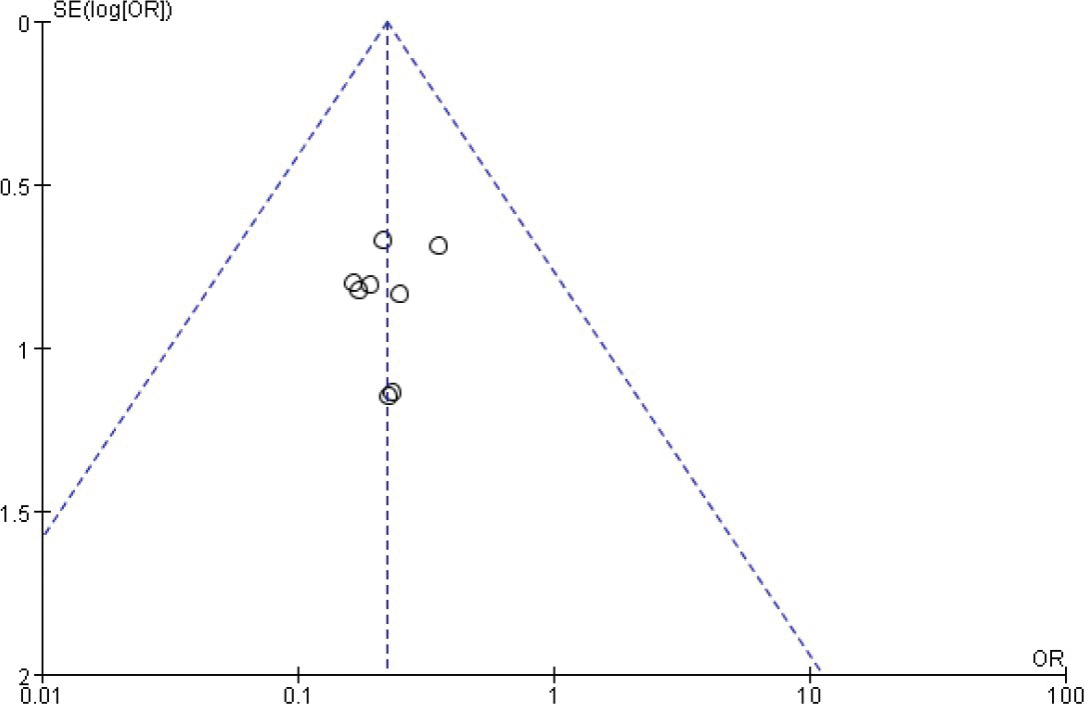
Funnel chart based on the incidence of adverse reactions.

The funnel chart was drawn based on success rate of repair, bone graft thickness, osteogenic thickness, histological level, and incidence of adverse reactions, and was used to analyse the publication bias (Figures 11–17). A few funnel charts asymmetries indicate publication bias, which may be due to study heterogeneity and limited number of examples included.

## Discussion

The findings of this systematic review and meta-analysis provide valuable insights into the efficacy and safety profiles of absorbable versus non-absorbable oral barrier membranes in guiding bone regeneration for dental implants. The results demonstrate that absorbable membranes, particularly collagen-based types, exhibit superior outcomes in several key aspects compared to their non-absorbable counterparts.

The analysis revealed a statistically significant higher success rate of repair in the absorbable membrane group (*p* < 0.05). This finding underscores the clinical advantages of absorbable membranes, which not only provide effective bone regeneration but also reduce the need for secondary surgical interventions. Secondary surgeries required for the removal of non-absorbable membranes, such as titanium-based barriers, can potentially contribute to complications and patient discomfort, which may negatively impact overall success rates. In the course of time, the artificial root will be closely combined with alveolar bone [44]. However, some patients cannot achieve satisfactory results due to various reasons. The application of GBR can improve the surgical effect, mainly according to the different movement speeds of fibroblasts, epithelial cells, and osteoblasts. The barrier membrane covers the bone defect and ensures the formation of a closed microenvironment, which in turn promotes tissue growth [45, 46].

Both bone graft thickness and osteogenic thickness were significantly higher in the absorbable membrane group (*p* < 0.05). These findings suggest that absorbable membranes create a more conducive environment for osteoblast activity and bone remodelling. The natural degradation process of collagen membranes ensures a sustained release of biomolecules that promote osteogenesis, thus enhancing the overall bone regeneration process. In contrast, the rigid structure of non-absorbable membranes may hinder optimal cellular migration and integration [47]. Biofilm barriers can play a key guiding role in dental implant bone regeneration, and it is clinically found that this technique can also increase the thickness of cementum. Biocompatible materials are used in oral barrier membranes, which are surgically placed between oral soft tissue and bone defects in order to create a relatively closed environment in which bone regeneration can occur. Fibroblasts and epithelial cells with a faster migration capacity are selectively prevented from entering the bone defect area without impeding the natural healing of the wound [22, 48]. PRF membranes, as an autologous biomaterial, are widely used in regenerative medicine and dentistry to facilitate tissue repair. Various types of PRF, including L-PRF (Leukocyte- and PRF), A-PRF (Advanced PRF), and A-PRF+ (Advanced PRF Plus), are produced using different centrifugation speeds and durations. Numerous studies have investigated the mechanical and structural properties of these membranes. For example, a study comparing the tensile strength of three types of PRF membranes (L-PRF, A-PRF, and A-PRF+) demonstrated that A-PRF+ exhibited the highest maximum tensile strength among them, indicating its superior viscoelastic resistance [49]. In addition, another study comparing the tensile strength between two types of autologous platelet concentrates (L-PRF and A-PRF) showed that A-PRF had both higher maximum and average tensile strength compared to L-PRF, indicating its greater resistance to opposing forces [50].

The histological analysis, including osseointegration, total mineralised tissue, and non-mineralised tissue, did not reveal statistically significant differences between the two groups (*p* > 0.05). This result suggests that while both membrane types are effective in promoting osseointegration, the choice of membrane might be influenced more by factors such as patient-specific needs, cost, and risk of complications, rather than their direct impact on histological parameters. The use of oral barrier membranes in periodontal mucosa surgery, implant surgery, alveolar surgery, and other dental fields is widespread [45]. It not only solves a large number of oral restoration problems but also meets the requirements of oral aesthetics. However, oral barrier membranes also have some problems, such as short maintenance time, antigenicity, soft tissue infection, membrane collapse, exposure, and infections, which need to be further studied and improved [22, 45]. The application of GBR can improve the above-stated problems and enhance the regeneration and healing of alveolar bone injuries.

One of the most compelling advantages of absorbable membranes highlighted in this study is their significantly lower incidence of adverse reactions (*p* < 0.05). The risk of complications such as dehiscence, infection, and wound exposure is markedly reduced with absorbable membranes. This can be attributed to their superior biocompatibility and natural degradation, which eliminates the need for membrane retrieval and minimises the risk of inflammatory responses. The degradation of materials in oral barrier membranes can be divided into absorbable and non-absorbable types. The production of GBR membranes, including both titanium and collagen, has evolved significantly over time. Titanium membranes are typically fabricated using advanced 3D virtual planning and precision manufacturing techniques, ensuring they fit the specific bone defect area for optimal mechanical support. Collagen membranes, on the other hand, are bioengineered from acellular xenogeneic or allogenic tissue, and are designed to degrade gradually, promoting tissue regeneration and integration with the surrounding bone and soft tissue without the need for secondary surgery [22]. Among them, titanium film, as a protective barrier, has a harder texture. Although it can form a closed environment and maintain better space and resistance strength, it is non-absorbable. This greatly limits blood entry into the bone graft site, hindering blood absorption and adversely affecting recovery [51]. The absorbable membrane, with characteristics like degradability, no cyto-toxicity, and longer degradation time, ensures the stability of blood clots and promotes wound healing and the combination of autogenous bone and new bone.

The findings of this review have important implications for clinical practice. Absorbable membranes are particularly advantageous in cases where patient compliance is a concern or where minimising surgical interventions are prioritised. However, the choice of membrane should also consider individual patient factors, such as the extent of bone defect, overall health status, and financial considerations. Some studies have shown that absorbable barrier membranes can not only increase the growth rate of bone grafts but also promote the combination of biofilm and new bone tissue, achieving the purpose of promoting the integration of autogenous bone and new bone [51]. Currently, a meta-analysis has shown that resorbable membranes are more effective when used to guide tissue regeneration for horizontal bone filling when treating root bifurcation lesions. Resorbable membranes are also more efficient than open flaps at reducing vertical detection depth, determining vertical attachment levels, and identifying horizontal and vertical bones [51, 52]. However, there are few meta-analyses on the clinical efficacy of bone regeneration guided by absorbable and non-absorbable oral barrier membranes.

Despite the robust methodology employed in this review, several limitations warrant consideration. Firstly, the heterogeneity among included studies in terms of intervention protocols and follow-up durations may influence the generalisability of the findings. Secondly, the relatively short follow-up periods in some studies limit the ability to assess long-term outcomes. Lastly, publication bias, as indicated by asymmetry in some funnel plots, may have impacted the comprehensiveness of the analysis.

Future research should aim to address these limitations by conducting high-quality, randomised controlled trials with standardised protocols and extended follow-up periods. In addition, investigating the cost-effectiveness and patient-reported outcomes associated with different membrane types could provide a more holistic understanding of their clinical utility.

## Conclusion

In conclusion, absorbable membranes offer a favourable balance of efficacy and safety in guiding bone regeneration for dental implants, making them a preferred choice in many clinical scenarios. However, the selection of the appropriate membrane type should be tailored to individual patient needs and clinical settings. Ongoing advancements in membrane materials and fabrication technologies hold promise for further enhancing the outcomes of GBR. These findings may encourage broader clinical adoption of absorbable membranes and PRF, particularly among practitioners who have not yet integrated PRF into their regenerative protocols.

## Declarations

### Availability of data and materials

All data generated or analysed during this study are included in this published article.
